# Computational Analysis of the Ligand-Binding Sites of the Molecular Chaperone OppA from *Yersinia pseudotuberculosis*

**DOI:** 10.3390/ijms24044023

**Published:** 2023-02-16

**Authors:** Mirian Becerril Ramírez, Lucía Soto Urzúa, María de los Ángeles Martínez Martínez, Luis Javier Martínez Morales

**Affiliations:** 1 Centro de Investigaciones en Ciencias Microbiológicas, Instituto de Ciencias, Benemérita Universidad Autónoma de Puebla, Puebla CP 72570, Mexico; 2 Facultad de Medicina, Benemérita Universidad Autónoma de Puebla, Puebla CP 72410, Mexico

**Keywords:** *Yersinia pseudotuberculosis*, OppA protein, interactions between OppA and ligands, chaperones

## Abstract

The function of chaperones is to correct or degrade misfolded proteins inside the cell. Classic molecular chaperones such as GroEL and DnaK have not been found in the periplasm of *Yersinia pseudotuberculosis*. Some periplasmic substrate-binding proteins could be bifunctional, such as OppA. Using bioinformatic tools, we try to elucidate the nature of the interactions between OppA and ligands from four proteins with different oligomeric states. Using the crystal structure of the proteins Mal12 alpha-glucosidase from *Saccharomyces cerevisiae* S288C, LDH rabbit muscle lactate dehydrogenase, *Eco*RI endonuclease from *Escherichia coli* and THG *Geotrichum candidum* lipase, a hundred models were obtained in total, including five different ligands from each enzyme with five conformations of each ligand. The best values for Mal12 stem from ligands 4 and 5, with conformation 5 for both; for LDH, ligands 1 and 4, with conformations 2 and 4, respectively; for *Eco*RI, ligands 3 and 5, with conformation 1 for both; and for THG, ligands 2 and 3, with conformation 1 for both. The interactions were analyzed with LigProt, and the length of the hydrogen bridges has an average of 2.8 to 3.0 Å. The interaction within the OppA pocket is energetically favored due to the formation of hydrogen bonds both of OppA and of the selected enzymes. The Asp 419 residue is important in these junctions.

## 1. Introduction

All organisms are endowed with complex attack, regulation or defense mechanisms that allow us to deal with day-to-day conditions in our environment. These systems are not only normal, but are necessary for survival. It is known that proteins are the main components of an organism’s structural, enzymatic, hormonal, protective, transport, and homeostatic functions, among others [1]. Proteins must obtain three-dimensional structures to perform their biological functions; however, environmental stress factors promote protein unfolding [2,3]. Unfolding proteins interact with themselves or other unfolding proteins, by means of their exposed hydrophobic regions, to form aggregates which are harmful to cells [4,5].

Adaptation to the environmental conditions is essential for survival; therefore, there are protein systems responsible for ensuring the proteome’s proper functioning [2,6]. Chaperones are folding control centers [7], some of which are produced in response to stress to correct or degrade misfolded proteins within the cell and ensure their correct activation, avoiding the risk of misfolding on themselves or with others and forming aggregated toxic species [8].

*Yersinia pseudotuberculosis* is a Gram-negative coccobacillus, it is one of the three pathogenic strains for humans, and it can grow in a large range of temperatures, from 4 to 40 °C. It has a tropism for lymph nodes, and it has a typical cell wall. Its virulence factors depend on the presence of the characteristic virulence plasmids of the genus, whose expression is strongly influenced by temperature, so adaptation to this stress factor is essential [9,10]. In its periplasmic space are phosphatases, proteases and endonucleases, enzymes for antibiotic resistance, importers, and exporters, among other proteins of diverse function, which are more exposed to external environmental stress than their cytoplasmic counterparts [11]. It seems to be necessary to have a misfolding preventive mechanism, such as the chaperon-based refold mechanism, to avoid the potential toxic aggregates produced by accelerated or uncontrolled protein denaturation [12,13].

Proteostasis comprises correct protein synthesis, folding, unfolding and turnover. It is mediated by chaperone and protease systems, along with autophagy and lysosomal degradation processes [4,7]. Some chaperonins belong to a group of heat shock proteins with an aggregation inhibitory function, which influence the intramolecular interactions that define the folding pathway of a protein by binding and protecting hydrophobic regions in non-native protein conformations [5]. They are known to be conserved in all organisms and are organized according to their molecular weight, and to bind to their ligand with weak hydrophobic and transient hydrogen bonds, which are soon overcome by the intramolecular forces of the ligand, released after a complete protein refolding.

To limit the number of chaperones without compromising the correct functioning and stability of the proteome, they have vast association versatility with different ligands [14], as classical molecular chaperones such as GroEL and DnaK do, but these chaperonins have not been found in the periplasm of *Yersinia pseudotuberculosis*, and this may be because this compartment has different conditions than the cytoplasm [5]. However, a new class of periplasmic proteins with chaperone-like activity has recently emerged, including the histone-like protein *E. coli* Skp, the rotamass, PpiD, SurA and FkpA, and the protease DegP / DsbG. This is an indication that some proteins have a dual function, with stable or even elevated translation under stress compared to other cytoplasmic proteins [14,15]. With that, it has been shown that some periplasmic proteins of different kinds have dual functionality, all of them demonstrating chaperone-like properties.

ATP-binding cassette (ABC) transporters constitute a superfamily of integral membrane proteins responsible for the ATP-driven translocation of many substrates. It is characterized by the presence of a P-type trafficking ATPase, comprising two cytosolic nucleotide-binding domains (NBDs) and two transmembrane domains (TMDs) [16,17]. Type I and II ABC importers rely on additional soluble substrate-binding domains (SBDs) or substrate-binding proteins (SBPs), which capture the transported substrate on the trans side and deliver it to TMDs [16,17]. Some periplasmic substrate binding proteins are abundant and have also been studied according to the presumption that they have bifunctional characteristics [18].

A few of Opp enzymes also play a role in other cellular activities, such as signal transduction, motility, respiration [19], regulation of gene expression, chemotaxis, competencies development, sporulation, conjugal DNA transfer, virulence development [20], metabolism of cell wall peptides, cell division [21], and as a molecular chaperone to help proteins fold in the periplasmic space. In the same way, the SBP subunit from the Opp oligopeptide transport system, which is responsible for initial substrate recognition and binding to provide nutrition to cells, can also have multiple roles on its own [22,23]. As we mentioned, general molecular chaperones seem non-existent in the periplasmic area, and other diverse proteins have been shown to have a dual function. The process of proteostasis in this zone is not well understood; however, components of ABC-type transporters with the ability to refold in stress situations have been identified [24,25]

Naturally, it can be said that a chaperone has the versatility of binding to a wide range of ligands, because otherwise it would be a very large energy expenditure for the microorganism to generate a chaperone for each ligand. [26]. The SBPs have great union versatility with different ligands too. OppA can bind chains of up to 35 residues, but it prefers polypeptides longer than five residues [27]. In addition, multiple OppA conformations can activate transport; nevertheless, but not all conformational states of SBP seem to provide the signal to facilitate transport, and only a single closed conformation of the SBP can interact with the translocating portion [27]. Other studies also suggested the protective role of OppA overexpression against heat and salt stress [28], as a major virulence determinant, and an inhibitor of complement deposition [29]. In the same way, it has been demonstrated involvement in terms of competence and sporulation [30], adhesion to host cells and periplasmic stress protection [26,31].

It has been shown that the α-amino group of the ligand is anchored through an ion-pair interaction with Asp 419, while the α-carboxylate group forms varied water-mediated interactions with key binding site amino acid residues: Glu32, Val34, Tyr109, Asn246, Asn247, Arg404, His405, Arg413, Ala415, Trp416, Cys417, Asp419 and Thr438. They all belong to the hydrophobic cleft near the hinge region [25].

Chaperone-like activity for OppA from *Yersinia pseudotuberculosis* has been observed in the laboratory when paired with partially denatured α-glucosidase and LDH-A enzymes, which do not share any structural or functional characteristics [25]. In vivo studies have shown four residues (arginine 41 and aspartate 42; aspartate 419 and tyrosine 420) and the role they play during denatured protein docking at the site. By performing site-directed mutagenesis, changes to these residues were found to affect chaperone-like activity in OppA [25]. Therefore, it is important to know other binding sites and what ligands of these proteins are involved, to determine if OppA has a chaperone function on other enzymes.

Here, we will examine the possible response *Y. pseudotuberculosis* has to stress, through the Opp importer system receptor protein chaperone-like action. Using computational molecular docking, we will study the interactions between OppA and the ligand protein. We will analyze the binding affinity at the active site of the enzyme and to observe the type of binding formed with the ligands extracted from the protein model, among other characteristics of these amino acid sequences.

## 2. Results

**Figure 1:**
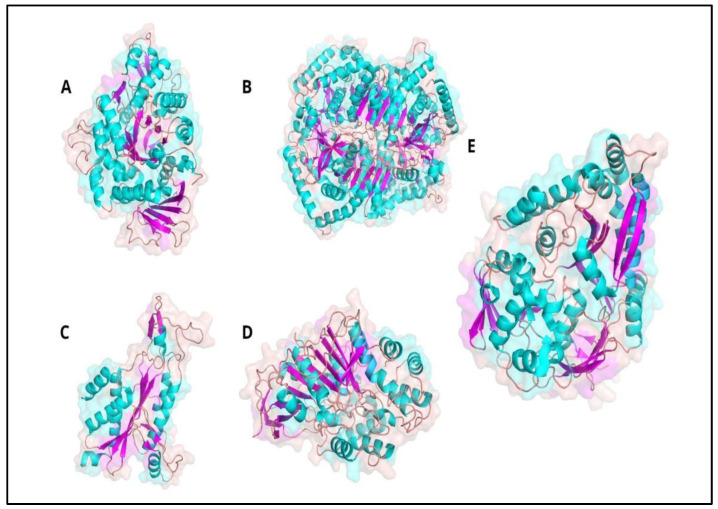
View of the OppA and its possible ligand crystal structures in the ribbon model used at this study. (**A**) Crystal structure of the alpha-glucosidase monomer from *Saccharomyces cerevisiae* S288C (Mal12). (**B**) Crystal structure of a tetramer of rabbit muscle *Oryctolagus cuniculus*, lactate dehydrogenase (LDH). (**C**) Crystal structure of the *E. coli* endonuclease dimer in complex with DNA chain (*Eco*RI). (**D**) Crystal structure of *Geotrichum candidum* lipase (THG). (**E**) Crystal structure of OppA from *Yersinia pestis*.

The current computational studies focus on the interaction of different ligands extracted from five different proteins, enzymes which have different structural and physicochemical characteristics. The aim is to examine the nature of the interactions that are formed between the OppA in its active site and the ligand (Protein Plus Visualizer, Figure 1).

**Figure 2:**
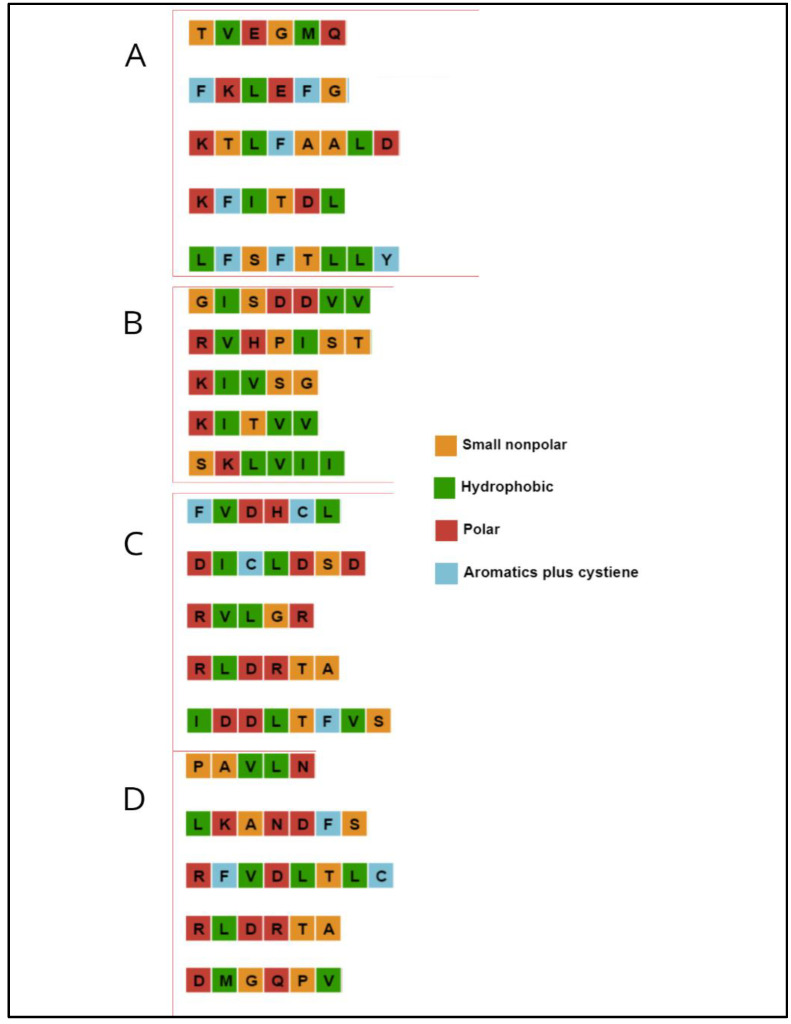
Graphic representation of the amino acid sequence of the ligands extracted from (**A**) the Mal 12 alpha glucosidase, (**B**) the A chain of the lactate dehydrogenase LDH, (**C**) *Eco*RI endonuclease and (**D**) THG lipase. The color code indicates the nature of the amino acid.

The protein data bank crystallographic structures were modified with Chimera 1.7 to obtain monomers only. With this, the initial docking was carried out with the complete proteins to evaluate the possible interaction zones from which the ligands would be extracted to perform a second more directed and closed docking. These areas of greatest interaction turned out to be the most hydrophobic—both the β-folded structures of the central barrel and the β-sandwich domains. The exact interphase residues with OppA were identified and the first five models were compared to select the stretches of common amino acid sequences. Five chains of between five and nine residues were selected, which were used as a template to create new structures and to be able to use the Avogadro program for the self-optimization of the ligand bond angles (Psipred server, Figure 2)

**Figure 3:**
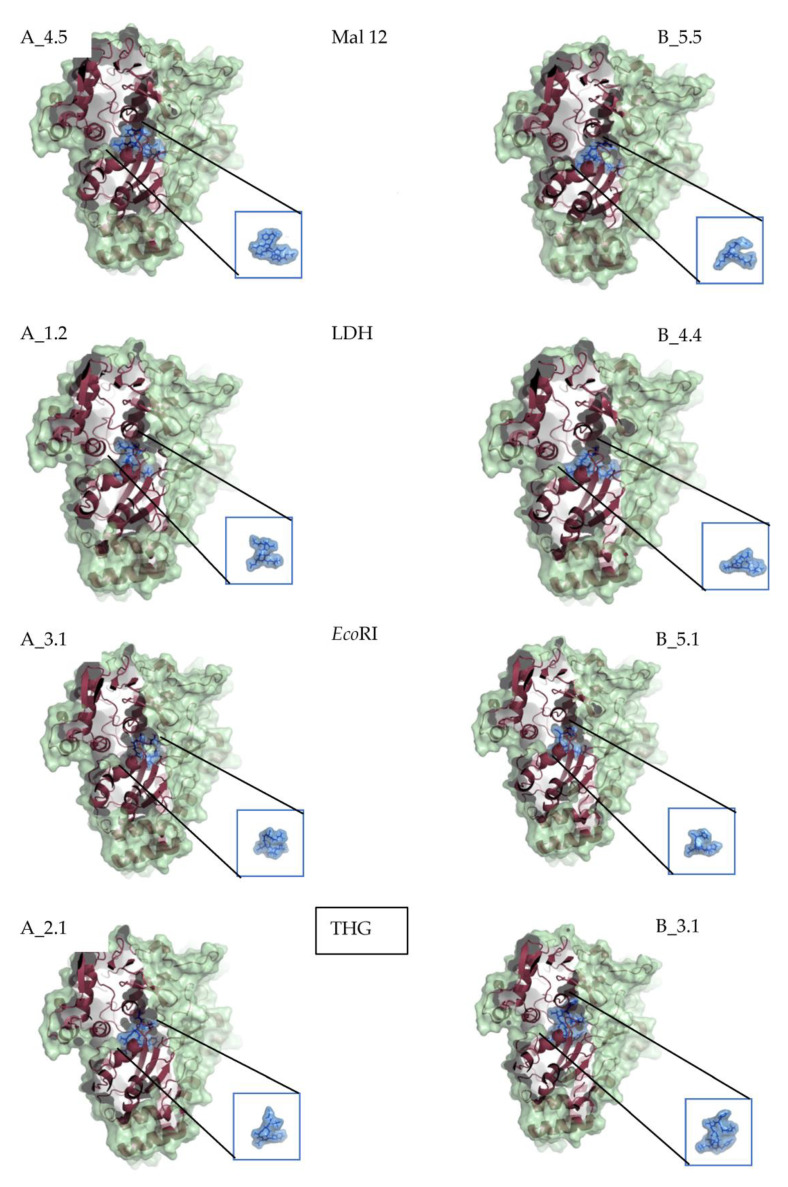
Positions of the best conformations of the two outstanding ligands of Mal12, LDH, *Eco*RI and THG. The colors in the OppA region indicate its surface shape (green), the ribbon representation of the protein (red) and the ligand (blue).

The OppA enzyme was chosen as a macromolecule and prepared for the studies; its ligand only bound to the active site of the enzyme. Hydrogen atoms were added as polar hydrogen. Subsequently, the general selection of the OppA was carried out. No seed was introduced, and the position and angle parameters were randomized. (Figure 3).

**Figure 4:**
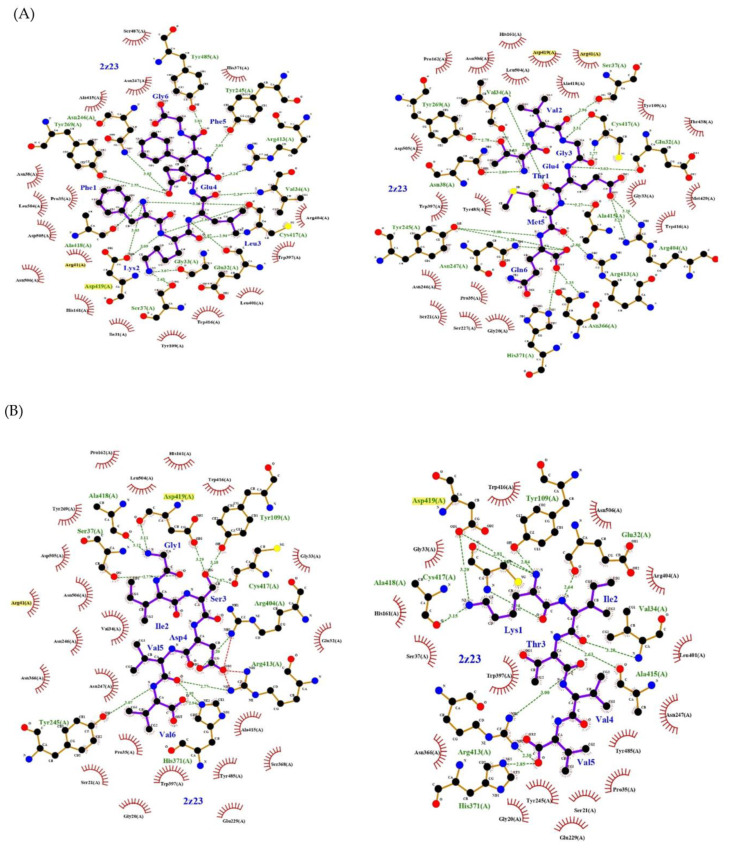

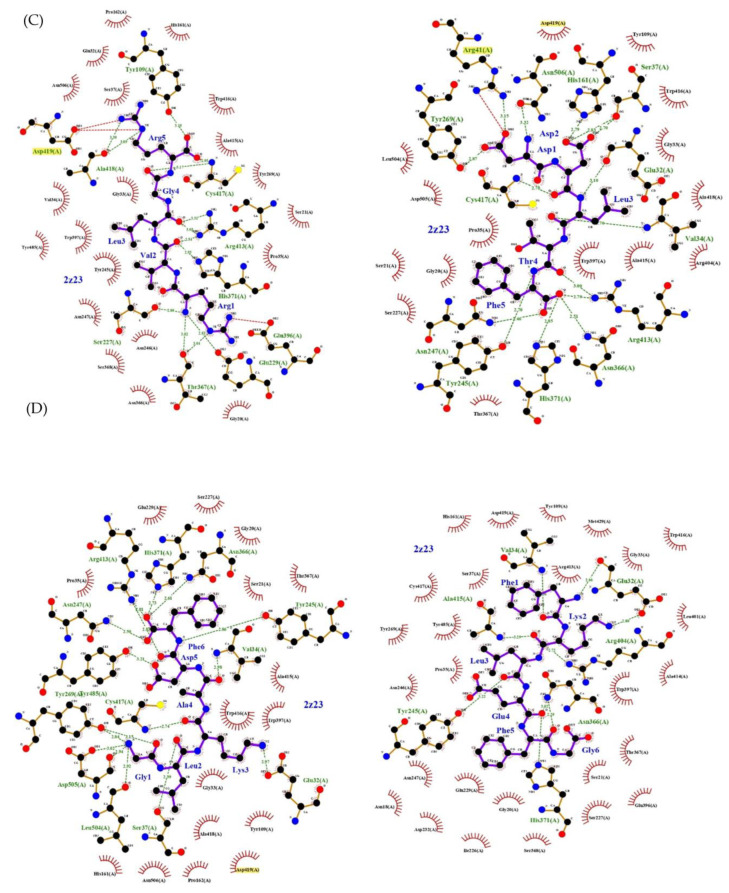
Two-dimensional diagrams, generated in LigPlot from the results of the molecular docking performed with Autodoock Vina, of the interactions of the two best ligands extracted from the structure of (**A**) the Mal12 hydrolase, (**B**) LDH lactate dehydrogenase, (**C**) *Eco*RI nuclease and (**D**) THG lipase. Hydrogen bonds formed between the amino acids of the protein (yellow) and its ligand (purple) can be seen as green dotted lines; salt bridges are depicted as red dotted lines; and hydrophobic interactions with the amino acid are delimited by a semicircle with red tabs. The distance between the links was calculated with LigProt.

Finally, the models with the best Vina scores for each ligand were selected, i.e., the binding energy (Kcal/mol) is higher the more negative this energy value becomes. Subsequently, the PDB files of each of the results were generated, and in this way, they could be subjected to more detailed analysis in the LigPLot program to verify the interactions through 2D schemes (Figure 4).

Most ligands coupled well with OppA, and at least one of the four amino acids of interest was present on all occasions. OppA prefers peptides with at least one arginine, histidine, or lysine. The identified binding candidates indicate that the peptide specificity of OppA is not limited to peptides of three to five amino acids; although good performance was demonstrated with these chain lengths, it should be noted that OppA can form interactions with positive residues and hydrophobic as well as longer peptides (Figure 2).

OppA has shown great specificity and stability with its ligands, which is due to hydrogen bonds between the peptide backbone and some other, less common, lateral interactions Table 1 and Supplementary Material. In addition, salt bridges can also be formed with residues with ionizable side chains such as serine, histidine, and tyrosine, but more commonly they are formed between the anionic atom of aspartate or glutamate and the cationic atom of lysine and/or arginine.

When a protein loses its native conformation, their hydrophobic motifs are buried and then exposed by the solvent, so OppA must recognize the unfolded structures and not those that maintain their three-dimensional conformation when interacting with residues with polar hydrophobic characteristics (Figure 3). These residues can find the necessary stability within the OppA pocket by forming hydrogen bonds. The activity of SBP would be reduced by acquiring an angle different from that required by the transmembrane motifs of the transporter, thus leaving the alternation to its chaperone activity [4].

All eight ligands bind to hydrophobic zones of the corresponding enzymes, mainly beta-pleated structures, which usually have the characteristic of being buried in the protein and generate internal hydrophobic interactions (from Mal12: V, M and L; from LDH: I, V and L; from *Eco*RI: I, L and V; from THG: V, L and M) to provide stability to the protein and prevent interactions in its hydrophilic part which generate its splitting in combination with average hydrogen bonds of 2.8 to 3.0 Å, allowing the interaction to be flexible (Figure 4).

The results suggest that the hydrophobic amino acids buried in the mostly hydrophilic structures of the SBP bind relatively strongly to hydrophobic residues of non-native proteins, generating patches of stable and non-specific interaction between the binding proteins and the unfolded proteins. Three-dimensional models reveal how peptides are accommodated within OppA and show that the lengths of oligopeptides tested in this study can be fully enclosed in the binding protein cleft (Figure 3).

## 3. Discussion

Proteins are limited and are needed in a wide variety of processes. The energy cost to maintain a group of proteins as diverse as the reactions they carry out would be very high [32], so it is natural to consider a certain multifunctionality of different types of proteins. In addition to the aforementioned observations, the folding process carried out by heat shock proteins within the cytoplasm or in cellular organelles is not applicable to the one that could exist in the extracytoplasmic environment. The periplasm of Gram-negative bacteria is not adequate for classical chaperones, since it has peculiar conditions. In this area, there are no ATP molecules, it is a highly oxidizing environment, and it is separated from the outside by only a fine porous membrane; therefore, it is especially susceptible to outside changes. [33,34].

The proteins with periplasmic chaperone activity, such as SurA, Skp, LolA, DegP, HdeA and Spy, [18] can play a role as proteases or facilitators in the transport of proteins to the outer membrane, allow the dimerization of a protein [35], or even play a role in the folding of β-barrel structures [15,36]; however, their main function is not the renaturation of proteins denatured by stress factors, and the synthesis of these proteins is decreased due to the activation of the stress response [37]. The bioinformatic evidence shown here proposes that the OppA periplasmic substrate-binding protein, from *Yersinia pseudotuberculosis*, in addition to serving the OppABCDE transport system, has a chaperone-like function, and participates in protein partially denatured refolding, recognizing its client protein through the uncovered sequences due losing its native conformation.

SBPs have been found to undergo a conformational change upon binding to their substrate; however, their ligand-binding site for their chaperone activity is not involved in this interaction [26], so it can be said that the conformational change induced by preferred ligands does not influence SBPs’ interaction with unfolded proteins, nor their binding to the transmembrane portion B and C of the system, which would suggest that the residues involved in the interaction with the ligand, the binding to the TMD portion and those that are mostly involved in the protein renaturation activity are different.

Only one of the four amino acid residues found in the results of the research carried out in 2020 by Escobar Garduño [25] repeatedly appeared to generate hydrogen bonds with the peptide ligand therefore, it seems that the other three residues of interest could be more directly related to a function within the flexibility of OppA.

Residue 419 was that which appeared the most in the interactions; however, in Escobar’s study [25], it did not seem to be important in the folding process, since the mutations generated from said amino acid did not generate changes in the refolding levels of the alpha glucosidase and lactate dehydrogenase. This may indicate that its main function could be in the generation of interactions, while the pair 41-42 could presumably be more linked to the flexibility of OppA, and thus mutations in these sites could result in less flexible conformations of the SBP in question, so that its chaperone activity would be clearly diminished by not having the opening capacity required for coupling. In all the docking tests that were carried out between OppA and different ligands, the 41-42 pair did not appear to form bonds. With this and the information on the functioning of these chaperonins, it can be inferred that they play another important role within OppA.

Spy has been shown to have the ability to bind both folded and partially denatured native proteins with its positive residue binding site and allows their folding. Spy’s protein-bound structure does not undergo major alterations, other than an increase in its flexibility. Surprisingly, it was found that Spy’s chaperone activity can be increased up to seven-fold with a more flexible mutant. Therefore, it is inferred that flexibility is of paramount importance for protein–ligand coupling [18].

The interactions between the chaperone and the ligand proteins involve hydrophobic and hydrophilic residues. Therefore, the properties of the folding state are essential for the establishment of the interaction, while the properties of the simple sequence do not seem to have an impact on chaperone-type interactions, as they might have on a carrier-type interaction. For OppA from *S. typhimurium*, the residue that interacts with the α-amino group of its ligand is an aspartate at position 419, as appears to be the case with OppA from *Y pseudotuberculosis*. In *E. coli* SBP DppA, with which OppA can share ligands, the residue that interacts with the α-amino group is also an aspartate at position 408, which is very close to Asp 419 of OppA [38].

The structures of OppA from other microorganisms are similar; however, the ability to bind with peptide chains of more than five units has not been proven, in these studies, the preference characteristic that OppA has towards Lysines is taken into account, for the generation of the ligands used in their molecular couplings, but said ligands do not come from a bacterial protein. In our study this preference is not taken into account and we focus on analyzing the possible interactions between OppA with peptides extracted from microbial enzymes, two of which have already been tested in the laboratory by Escobar. Our ligands, in addition to belonging to biological sequences, are greater than five units and seem to fit perfectly into the OppA pocket of *Y. pseudotubeerculosis* with high affinity and a high number of interactions [22,39,40,41,42]

Most SBPs are known to have low-level expression in the absence of their specific inducer; in contrast, OppA is constitutively expressed in considerable amounts [43]. Under this precept, together with the fact that it is thermostable and its presumed plasticity of function, it could be considered as an indicator that this type of protein has a prominent role as chaperones. The SBPs OppA and MalE from *E. coli* and MglB from *Salmonella typhimurium* have shown a chaperone-like function. Their synthesis does not increase after raising the temperature—it remains constant, unlike other cellular proteins, which are seen diminish. Apart from that, they were shown to be able to renature relatively similar percentages of active protein compared to DnaK and DnaJ [richarme].

In addition, OppA from *E. coli* has been shown to bind to the permanently unfolded protein R-CMLA, in a similar percentage to DnaK. With the above, it is important to consider that OppA at least shares its binding characteristics with those of the unfolded proteins that chaperones have. Chaperones can bind a wide variety of ligands, although it has certainly been found that each one has a predilection for certain molecules. This phenomenon of selection of a protein for binding to a ligand has been best studied in those that have a role in import–export, or chemotaxis. To carry out the transport of the molecules, the SBPs must interact specifically with the transmembrane proteins that generally form a complex of two proteins, to which they must deliver the ligand.

In studies carried out in OppA of *S. typhimurium*, they reported that peptide chains that exceeded five amino acid residues would be destined to partially protrude from the binding protein [44]. However, in the bioinformatic work carried out here, it was observed that the OppA of *Y. pseudotubeculosis* is capable of completely enclosing oligopeptides of up to nine residues. It would be necessary to carry out more tests to know what would happen with peptides of greater lengths, in addition to seeing if this capacity would influence refolding proteins with a higher degree of misfolding.

## 4. Materials and Methods

The crystal structures used for this study with a similar resolution were extracted from Protein Data Bank: OppA from *Yersinia pestis* (PDB code: 2Z23) [45], α-glucosidase from *Saccharomyces cerevisiae* (PDB code: 3aj7) [46], rabbit muscle LDH-A (PDB code: 6P6U) [47], *Eco* RI endonuclease (PDB code: 1ERI) [48], and *Geotrichum candidum* lipase CRLc (PDB code: 1TRH) [49].

Four proteins were used. There were five ligands for each protein and five conformations for each ligand, giving a total of 100 different models. They all joined in a similar region of OppA, and the best models of each ligand were chosen, comparing the number of links, type of links, distance, and Vina score. To determine the sites of possible initial contact, protein–protein molecular docking was performed with the servers HDOCK and GRAMM-X [50,51]. From the location of the interaction sites, with the UCSF-Chimera program [52], five ligands of each model protein were selected and extracted: MAL-12, LDH-A, *Eco*RI and CRLc. To evaluate the interaction of the extracted oligopeptides with OppA, molecular docking was carried out under AutoDock Vina [53].

The analysis of ligand–receptor complex interactions and two-dimensional schemes of complexes with 3D structures were performed in LigProt software [54]. Lengths of hydrogen and saline bonds between 2.0 and 2.9 A were considered. Autodock required that the enzyme OppA was chosen as a macromolecule and prepared for the studies, where ligands, solvent molecules and other residues were removed from the active site of the enzyme. Hydrogen atoms were added to the structure with the Hydrogen Polar only option. Subsequently, for the grid box, a general selection of the OppA was performed. No seed was introduced, and the position and dihedral angle parameters were left random.

## 5. Conclusions

The interaction within the OppA pocket is energetically favored due to the formation of hydrogen bonds with those hydrophobic areas, both of OppA and of the selected enzymes.

Residue 419 is important for ligand binding and is not required for active folding. In addition, residues 41 and 42 do not appear in the binding with any ligand, but if they are involved in the chaperone-like activity, they match very well with the data from Escobar, 2020.

## Figures and Tables

**Table 1:** Conformation of ligands.

	Number of Ligands	Number of Conformations	Vina Score	Number Hydrogen Bonds	Number Salt Bridges	Average Hydrogen Bonds Distances in Å by LigProt	aa with Hydrogen Interactions
Mal 12	4	5	−7.3	15		2.8	TYR_485, TYR_245, ARG_413
	5	5	−9.4	12		2.8	TYR_109, CYS_417, ALA_415
LDH	1	2	−8	11	3	3.0	ASP_419, TYR_109, CYS_417
	4	4	−7.8	12		2.8	TYR_109, GLU_32, VAL_34
*Eco*RI	3	1	−7	13	3	2.9	TYR_109, CYS_417, ARG_413
	5	1	−9.6	16	1	2.9	TYR_269, ARG_41, ASN_506
THG	2	1	−7.7	14	2	2.9	HIS_161, CYS_417, ARG_404
	3	1	−8.2	9		2.8	GLU_32, ARG_404, ASN_366

Conformation 5 of ligands 4 and 5; conformations 2 and 4 of ligands 1 and 4; conformation 1 of ligands 3 and 5; and conformation 1 of ligands 2 and 4, extracted from Mal12, LDH, *Eco*RI and THG, respectively, have the best performance among the 5 initially proposed for each protein, since the correlations between the Vina score and the number of hydrogen bonds are favorable, being −7.3, −9.4/15, 16; −8, −7.8/11 + 3 salt bridge, 12; −7, −9.6/12 + 3 salt bridge, 15 + 1 salt bridge; −7.7, −8.2/13 + 2 salt bridge, 8; following the same order (more information in Supplementary Material).

## Data Availability

Not applicable.

## Supplementary Materials

The following supporting information can be downloaded at: https://www.mdpi.com/article/10.3390/ijms24044023/s1.
