# Supplementary material for: Computational Analysis of the Ligand-Binding Sites of the Molecular Chaperone OppA from Yersinia pseudotuberculosis
Source: Int J Mol Sci. 2023 Feb 16;24(4):4023. doi: 10.3390/ijms24044023 (PMC9967938; doi:10.3390/ijms24044023)
Supplement: Supplementary file 1 [file ijms-24-04023-s001.zip › ijms-2119095-supplementary.pdf]

Computational analysis of the interactions of OppA from *Yersinia pseudotuberculosis* and its ligands.

Mirian Becerril Ramírez[1], Lucía Soto Urzúa[1], María de los Ángeles Martínez Martínez[2] and Luis Javier Martínez Morales[1*]

[1]Centro de Investigaciones en Ciencias Microbiológicas, Instituto de Ciencias. [2]Facultad de Medicina. Benemérita Universidad Autónoma de Puebla. México.

Supplementary material

Figure S1
OppA interaction zones with four different enzymes (Mal12, LDH, *Eco*RI and THG)

MAL12 enzyme

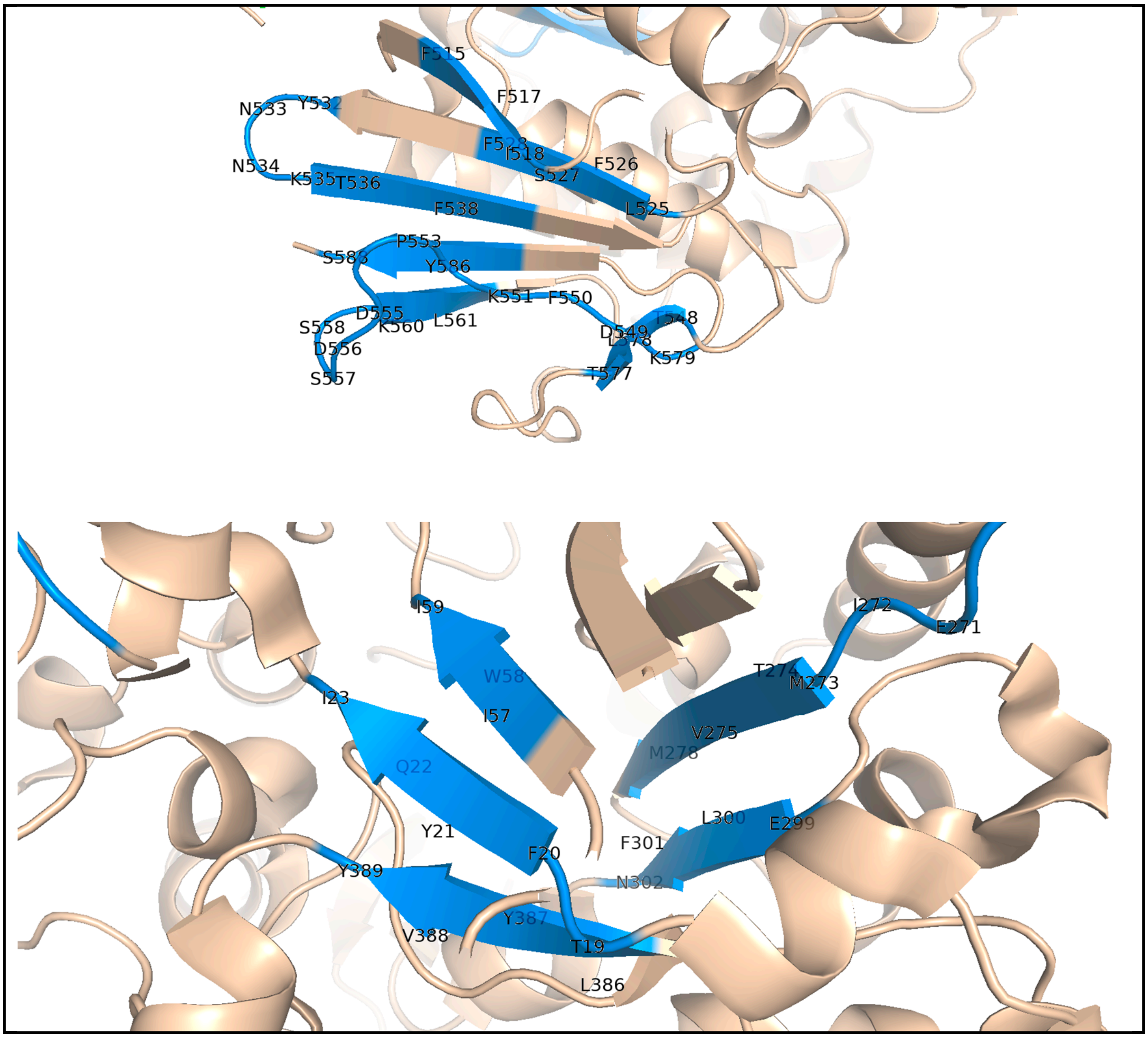

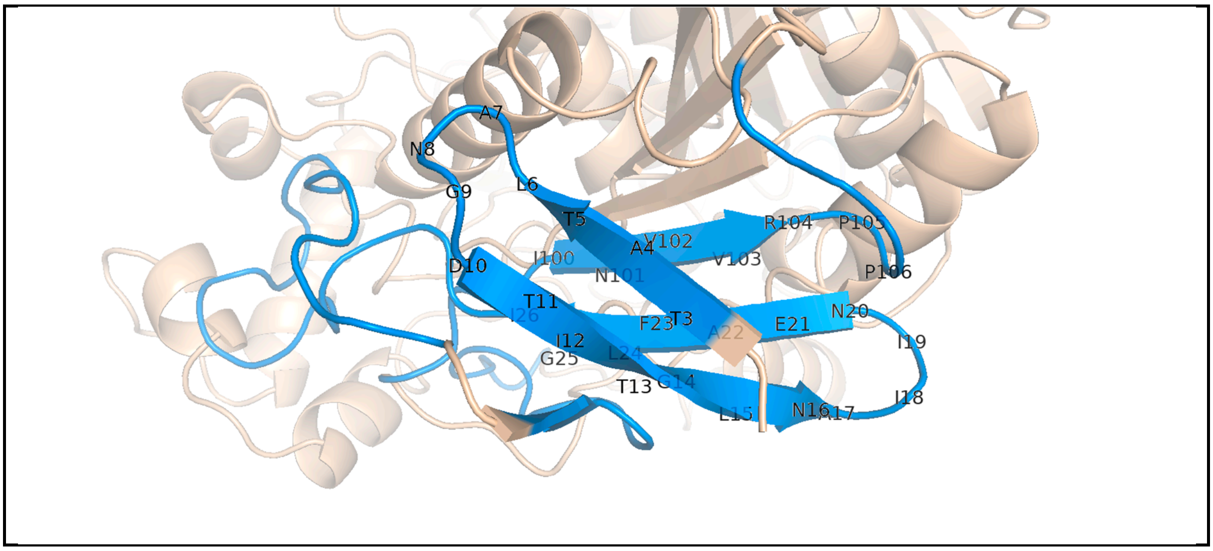
A7
N8
G9
L6
T5
D10
I100
A4
V102
N101
V103
R104
P105
P106
T11
I26
F23
T3
A22
E21
N20
I19
I12
G25
L24
T13
G14
L15
N16
A17
I18

LDH-A enzyme

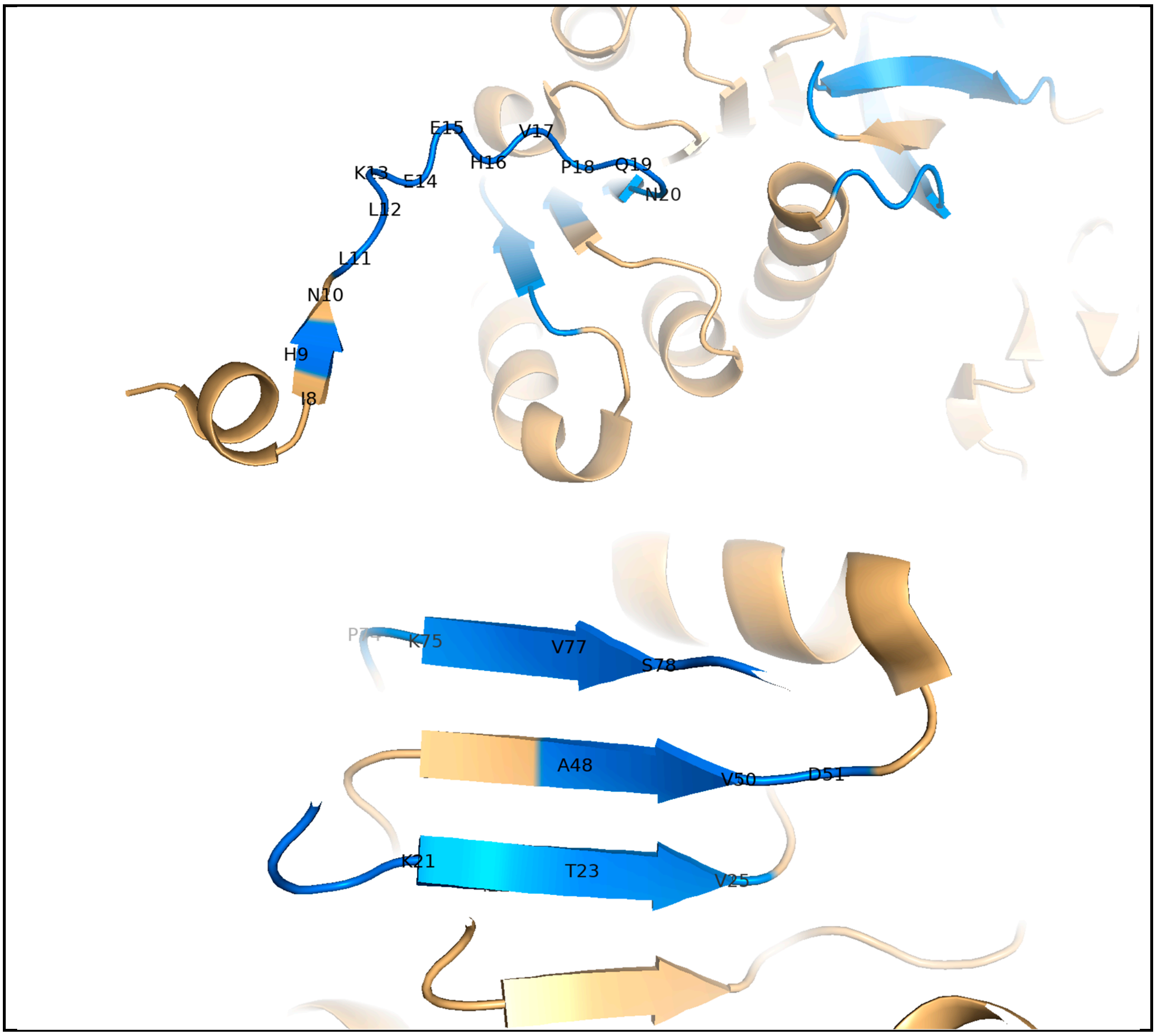
E15
V17
H16
K13
E14
P18
Q19
N20
L12
L11
N10
H9
I8
P74
K75
V77
S78
A48
V50
D51
K21
T23
V25

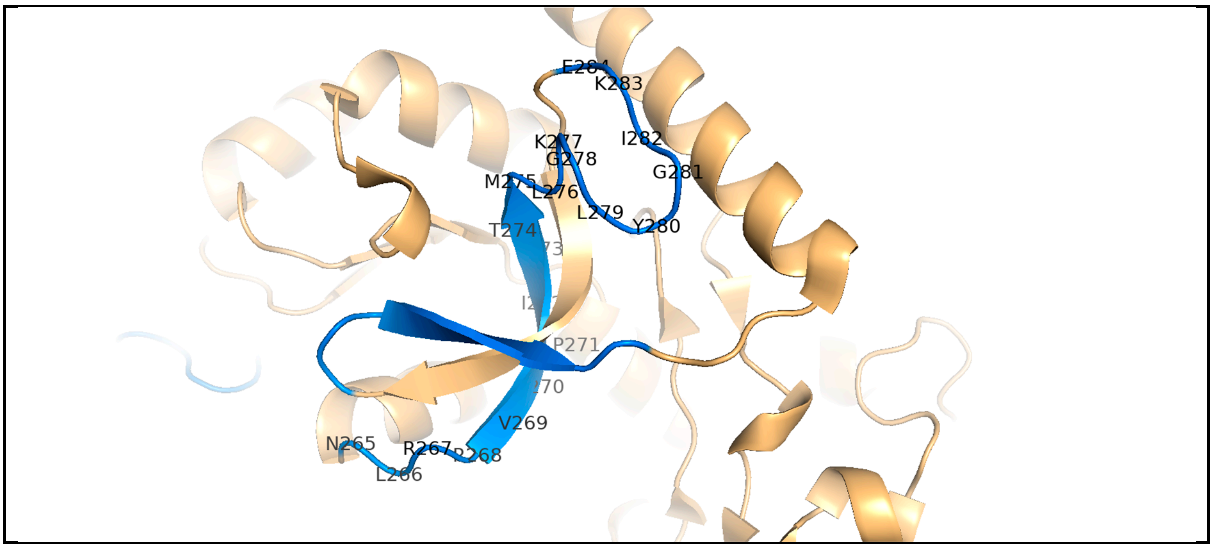

*Eco*RI enzyme

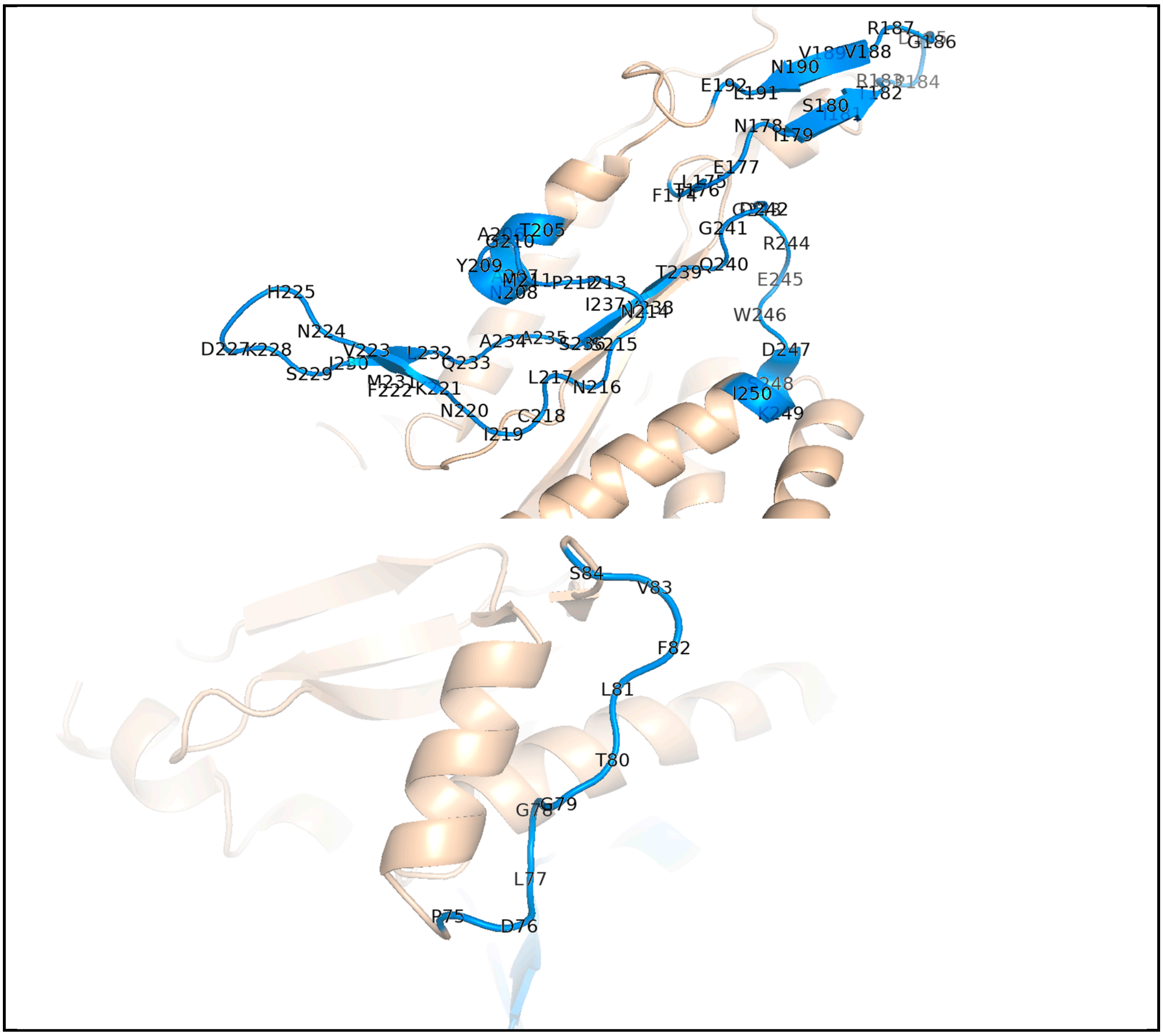

THG enzyme

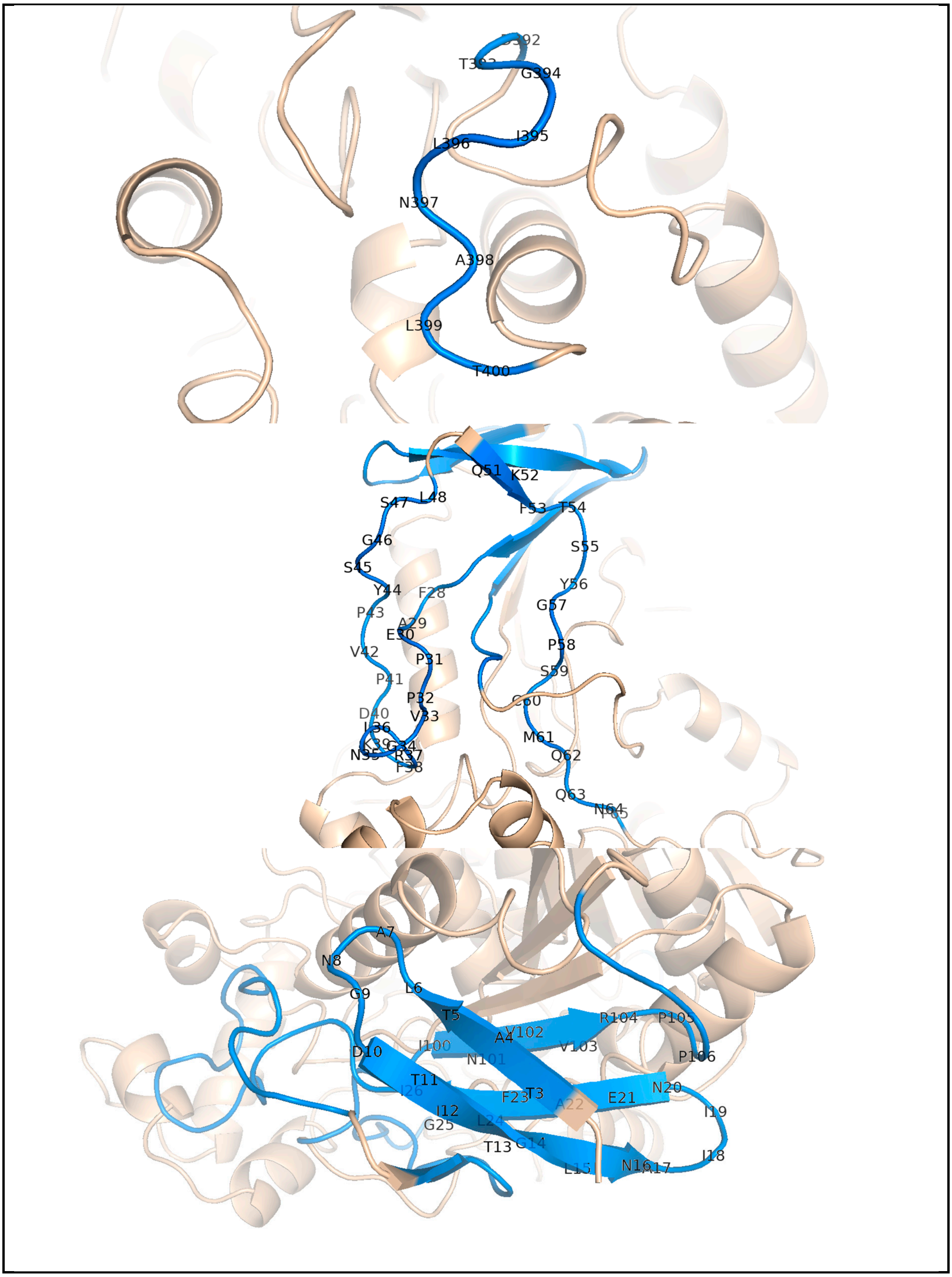
G394
I395
L396
N397
A398
L399
T400
Q51
K52
S47
L48
F53
T54
G46
S55
S45
Y44
F28
Y56
P43
A29
G57
E30
P58
V42
P31
S59
P41
P32
V33
D40
I36
M61
R37
Q62
F38
Q63
N64
A7
N8
L6
G9
T5
R104
P105
V102
A4
I100
V103
D10
N101
P106
T11
N20
F23
T3
A22
E21
I26
I19
I12
G25
L24
T13
G14
I18
L15
N16
A17

Table S1

HYDROGEN-BOND NETWORK AA

Enzime	OppA
Mal12_4.5	
PHE, LYS, LEU, GLU, PHE, GLY	TYR_485, TYR_245, ARG_413, VAL_34, CYS_417, GLN_32, GLY_33, SER_37, ALA_418, TYR_269, ASN_246
MAL12_5.5:	
THR, VAL, GLY, GLU, MET GLN	TYR_109, CYS_417, ALA_415, GLY_20, ASN_366, ARG_413, TYR_245, GLU_32, ASP_419
LDH_1.2	
GLY, SER, ASP, VAL, VAL	ASP_419, TYR_109, CYS_417, ARG_404, ARG_413, HIS_371, SER_37, ALA_418
LDH_4.4	
LYS, ILE, VAL	TYR_109, GLU_32, VAL_34, ALA_415, HIS_371, ARG_413, CYS_417, ALA_418, ASP_419
*Eco*RI_3.1	
ARG, VAL, LEU, GLY, ARG	TYR_109, CYS_417, ARG_413, HIS_371, GLU_396, GLU_229, THR_367, SER_227, ALA_418, ASP_419
*Eco*RI_5.1	
ASP, ASP, LEU, THR, PHE	TYR_269, ARG_41, ASN_506, HIS_161, SER_ 37, CYS_417, GLU_32, VAL_34, ARG_413, TYR_245, ASN_247, HIS_371, ASN_366, ARG_413
THG_2.1	
GLY, LEU, ALA, ASP, PHE	HIS_161, CYS_417, ARG_404, ALA_415, ARG_413, THR_367, ASN_366, SER_21, TYR_245, ASN_246, VAL_34, TYR_269
THG_3.1	
PHE, LYS LEU, GLU, PHE	GLU_32, ARG_404, ASN_366, HIS_371, TYR_245, ALA_415, VAL_34

Table S2

AA THAT HAS OPPA'S HYDROPHOBIC INTERACTIONS WITH Mal12, LDH, *Eco*RI and THG.

Enzime	OppA
Mal12_4.5	
PHE, LYS, LEU, GLU, PHE, GLY	SER_487, HIS_371, ASN_247, ALA_415, ASN_38, PRO_35, LEU_504, ASP_505, ARG_41, ASN_506, HIS_161, ILE_31, TYR_109, TRP_416, LEU_401, TRP_397, ARG_404
MAL12_5.5:	
THR, VAL, GLY, GLU, MET GLN	TRP_416, ASN_436, GLY_33, ARG_404, TRP_ 397, SER_227, SER_21, GLU_229, THR_367, SER_368, PRO_35, HIS_371, TYR_269, ASN_247, ASN_246, VAL_34, TYR_485, CYS_271
LDH_1.2	
GLY, ILE, SER, ASP, VAL, VAL	PRO_162, LEU_504, HIS_161, TRP_416, GLY_33, GLU_32, ALA_415, SER_368, TYR_485, GLU_229, TRP_397, GLY_20, SER_21, PRO_35, ASN_247, ASN_366, ASN_246, VAL_34, ARG_41, ASN_506, ASP_505, TYR_269
LDH_4.4	
LYS, ILE, THR, VAL, VAL	TRP_416, ASN_506, ARG_404, LEU_401, ASN_247, TYR_485, PRO_335, SER_21, GLU_229, TYR_245, GLY_20, ASN_366, TRP_397, SER_37, HIS_161, GLY_33
*Eco*RI_3.1	
ARG, VAL, LEU, GLY, ARG	PRO_162, HIS_161, TRP_416, ALA_415, TYR_269, SER_21, PRO_35, GLY_20, ASN_366, SER_368, ASN_246, ASN_247, TYR_245, TRP_397, TYR_485, VAL_34, GLY_33, SER_37, ASN_506, GLU_32
*Eco*RI_5.1	
ASP, ASP, LEU, THR, PHE	ASP_419, TYR_109, TRP_416, GLY_33, ALA_418, ARG_404, ALA_415, TRP_397, THR_367, SER_227, GLY_20, SER_21, PRO_35, ASP_505, LEU_504
THG_2.1	
GLY, LEU, LYS, ALA, ASP, PHE	ASP_419, SER_37, TRP_416, GLU_32, TYR_109, LEU_401, SER_227, GLY_20, HIS_371, SER_368, PRO_35, GLU_229, TRP_397, ASN_247, ASN_506, GLY_33, ALA_418, LEU_504
THG_3.1	
PHE, LYS LEU, GLU, PHE, GLY	ASP_419, TYR_109, ARG_413, MET_429, GLY_33, TRP_416, LEU_401, ALA_414, TRP_397, THR_367, SER_21, GLU_396, SER_227, SER_368, GLY_20, ILE_226, GLU_229, ASP_232, ASN_18, ASN_247,

	ASN_246, PRO_35, TYR_269, TYR_485, CLY_417, SER_37, HIS_161

Table S2

Hydrogen bond distance values and average (in Å) between OppA and Mal12, LDH, *Eco*RI, THG.

	1	2	3	4	5	6	7	8	9	10	11	12	13	14	15	16	Average
**Mal 12_4.5**	2.3	3.0	3.0	3.1	2.9	3.0	2.6	2.9	2.8	2.3	3.2	3.0	2.8	3.0	2.5		2.8
	Ala 418	Asp 419	Asp 419	Cys 417	Cys 417	Gly 33	Ser 37	Cys 417	Glu 32	Val 34	Arg 413	Tyr 245	Tyr 485	Asn 246	Tyr 269		
**Mal 12_5.5**	3.1	2.8	3.0	3.1	2.7	2.6	2.5	3.3	2.5	2.8	2.9	2.9					2.8
	Asp 419	Tyr 109	Cys 417	Cys 417	Glu 32	Ala 415	Tyr 245	Arg 413	Arg 413	Arg 413	Asn 366	Gly 20					
**LDH_1.2**	3.1	3.1	2.7	3.2	3.1	2.8	2.2	2.7	2.9	3.0	3.9						3.0
	Asp 419	Ala 418	Ser 37	Asp 419	Tyr 109	Cys 417	Arg 404	Arg 413	His 371	Tyr 245	His 371						
**LDH_4.4**	3.1	3.2	2.9	2.8	2.8	2.8	2.6	3.2	2.6	3.0	2.3	2.8					2.8
	Ala 418	Trp 416	Cys 417	Cys 418	Asp 419	Tyr 109	Glu 32	Val 34	Ala 415	Arg 413	Arg 413	His 371					
***Eco*RI_3.1**	3.0	2.8	3.0	2.8	2.9	2.5	3.0	3.3	3.1	2.9	3.1	3.0	3.0				2.9
	Thr 367	Glu 229	Thr 367	Ser 227	His 371	Arg 413	Arg 413	Arg 413	Cys 417	Cys 417	Tyr 109	Ala 418	Ala 418				
***Eco*RI_5.1**	2.8	3.1	3.3	2.7	2.8	2.7	2.7	3.1	3.7	3	2.7	2.4	2.8	2.5	2.7	3	2.9
	Tyr 269	Arg 41	Asn 506	His 161	Ser 37	Ser 37	Cys 417	Glu 32	Val 34	Arg 413	Tyr 245	Asn 247	His 371	Asn 366	Arg 413		
**THG_2.1**	2.8	2.2	2.9	2.8	3	3.2	3.1	2.1	3.0	3.0	3.0	3.0	3.1	3.2			2.9
	Thr 367	Asn 366	Ser 21	Tyr 245	Asn 246	Tyr 269	Arg 413	Arg 413	Val 34	Arg 404	Arg 404	Ala 415	Cys 417	His 161			
**THG_3.1**	2.0	2.8	2.8	2.7	3.2	3.2	3.0	3.7	2.2								2.8
	Val 34	Glu 32	Glu 32	Arg 404	Ala 415	Tyr 245	Asn 366	His 371	Asn 366								
